# An ethyl acetate fraction derived from *Houttuynia cordata* extract inhibits the production of inflammatory markers by suppressing NF-кB and MAPK activation in lipopolysaccharide-stimulated RAW 264.7 macrophages

**DOI:** 10.1186/1472-6882-14-234

**Published:** 2014-07-10

**Authors:** Jin Mi Chun, Kyoung Jin Nho, Hyo Seon Kim, A Yeong Lee, Byeong Cheol Moon, Ho Kyoung Kim

**Affiliations:** 1 Herbal Medicine Resources Group, Korea Institute of Oriental Medicine, Yuseongdae-ro 1672, Yuseong-gu, Daejeon 305-811, Republic of Korea

**Keywords:** *Houttuynia cordata*, Inducible nitric oxide synthase, Cyclooxygenase-2, Nuclear factor-κB, Mitogen-activated protein kinase

## Abstract

**Background:**

*Houttuynia cordata* Thunb. (Saururaceae) has been used in traditional medicine for treatment of inflammatory diseases. This study evaluated the anti-inflammatory effects of an ethyl acetate fraction derived from a *Houttuynia cordata* extract (HCE-EA) on the production of inflammatory mediators and the activation of nuclear factor-κB (NF-κB) and mitogen-activated protein kinases (MAPKs) in lipopolysaccharide (LPS)-stimulated RAW 264.7 macrophages.

**Methods:**

To measure the effects of HCE-EA on pro-inflammatory cytokine and inflammatory mediator’s expression in RAW 264.7 cells, we used the following methods: cell viability assay, Griess reagent assay, enzyme-linked immunosorbent assay, real-time polymerase chain reaction and western blotting analysis.

**Results:**

HCE-EA downregulated nitric oxide (NO), prostaglandin E_2_ (PGE_2_), tumor necrosis factor-α (TNF-α), and interleukin (IL-6) production in the cells, as well as inducible nitric oxide synthase (iNOS) and cyclooxygenase-2 (COX-2) expression. Furthermore, HCE-EA suppressed nuclear translocation of the NF-κB p65 subunit, which correlated with an inhibitory effect on IκBα (nuclear factor of kappa light polypeptide gene enhancer in B-cells inhibitor, alpha) phosphorylation. HCE-EA also attenuated the activation of MAPKs (p38 and JNK).

**Conclusions:**

Our results suggest that the anti-inflammatory properties of HCE-EA may stem from the inhibition of pro-inflammatory mediators via suppression of NF-κB and MAPK signaling pathways.

## Background

Inflammation has been implicated as a pathophysiological mechanism underlying many chronic diseases. Inflammatory responses and clinical symptoms are controlled through cytokines, nitric oxide (NO), and lipid mediators, including prostaglandins and leukotrienes produced by macrophages, neutrophils, and other inflammatory cells [1,2]. Activated macrophages play a particularly important role in the mediation of inflammation via the generation of tumor necrosis factor-α (TNF-α), interleukin-6 (IL-6), NO, and prostaglandin E_2_ (PGE_2_). Of these, NO, PGE_2_ are generated by inducible nitric oxide synthase (iNOS) and cyclooxygenase-2 (COX-2), respectively [3,4].

Overproduction of these assorted inflammatory mediators is involved in the pathogenesis of atherosclerosis, inflammatory arthritis, and cancer [5]. Thus, identification of natural product-derived extracts that inhibit the production of inflammatory mediators is extremely attractive in terms of the development of functional foods for disease treatment and/or prevention [6].

Many previous studies have shown that various phytochemicals exert anti-inflammatory effects by inhibiting the nuclear factor κB (NF-κB) signaling pathway [7-11]. NF-κB is a predominant transcription factor involved in the regulation of immune and inflammatory responses. The activation of NF-κB occurs via phosphorylation and degradation of IκB bound to NF-κB, resulting in the translocation of NF-κB into the nucleus to promote the expression of pro-inflammatory mediators (i.e., iNOS, COX-2, and certain cytokines) [12]. Mitogen-activated protein kinase (MAPK) signaling pathways also modulate inflammatory responses through the upregulation of cytokine expression [13]. At the molecular level, the chemopreventive activities of anti-inflammatory substances are often attributed to their ability to target the components of pro-inflammatory signaling pathways, especially those mediated by a panel of upstream kinases and transcription factors [14]. Therefore, NF-κB and MAPKs are critical targets for the actions of anti-inflammatory molecules.

*Houttuynia cordata* Thunb. (family Saururaceae) is a perennial food plant widely distributed throughout Southeastern Asia. This plant is a leaf vegetable that is used as a herbal tea, in salads, or cooked with other vegetables [15]. Also, It has been used in traditional medicine for treatment of suppuration, chronic bronchitis and pneumonia, otitis, and cystitis [16,17]. *H. cordata* contains a number of polyphenolic components and is characterized by various pharmacological functions, including antioxidant, anti-inflammatory, anti-tumor, and anti-allergy properties [18-22]. Recent reports have demonstrated that the volatile oil and supercritical extract constituents of *H. cordata* are acutely important for the mitigation of inflammation [20,23,24]. Another recent study demonstrated that the ethyl acetate fraction of a *H. cordata* extract exerted a protective effect against chemokine (C-C motif) ligand 4 (CCL4)-induced acute hepatotoxicity in mice [15].

Despite these encouraging studies, the cellular and molecular mechanisms responsible for the anti-inflammatory activity of *H. cordata* have yet to be elucidated. Therefore, the present study compared the anti-inflammatory actions of *H. cordata* extract (HCE) and with those of various HCE fractions, by measuring their ability to inhibit NO, PGE_2_, TNF-α, and IL-6 production. Next, we investigated the molecular mechanisms underlying the anti-inflammatory impact of the most efficacious fraction of HCE, the HCE ethyl acetate fraction (HCE-EA).

## Methods

### Chemicals and reagents

Lipopolysaccharide (LPS), bovine serum albumin (BSA), and other common chemicals were purchased from Sigma-Aldrich Chemical Co. (St. Louis, MO, USA). Primary and secondary antibodies for Western blotting analysis were purchased from Santa Cruz Biotechnology (Santa Cruz, CA, USA). ELISA kits were obtained from R&D Systems (Minneapolis, MN, USA). The RNA extraction kit was obtained from Qiagen (Hilden, Germany). The Nuclear Extract Kit was purchased from Active Motif (Carlsbad, CA, USA). Standard compounds for ultra-high performance liquid chromatography (UPLC) analysis of *H. cordata* were as follows: chlorogenic acid (Sigma-Aldrich, Steinheim, Germany), hyperoside (Carl Roth GmbH, Karlsruhe, Germany), and quercitrin (Wako Pure Chemical Industries Ltd, Osaka, Japan).

### Plant materials and sample preparation

*H. cordata* was purchased from Omniherb Co. (Yeoungcheon, Korea) and authenticated based on the macroscopic characteristics described by the Classification and Identification Committee of the Korea Institute of Oriental Medicine (KIOM). The committee was composed of nine experts in the fields of plant taxonomy, botany, pharmacognosy, and herbology. A voucher specimen (KIOM008013) was deposited in the herbarium of the Department of Herbal Medicine Resource at KIOM. *H. cordata* (3 kg) was extracted three times with 70% ethanol (with a 2 h reflux), and the extract was concentrated under reduced pressure and lyophilized. The resulting extract (663.1 g) was resuspended in water and partitioned sequentially with *n*-hexane, methylene chloride, ethyl acetate, *n*-butanol, and water, followed by *in vacuo* evaporation to yield the *n*-hexane fraction (HCE-Hx, 70.9 g), the methylene chloride fraction (HCE-MC, 17.9 g), the ethyl acetate fraction (HCE-EA, 24.6 g), the *n*-butanol fraction (HCE-BuOH, 96.6 g), and the HCE-water fraction (228.6 g).

### Cell culture

RAW 264.7 murine macrophages were obtained from the American Type Culture Collection (ATCC, Rockville, MD, USA) and maintained in Dulbecco’s modified Eagle’s medium (DMEM) supplemented with 5.5% fetal bovine serum (FBS) and 1% penicillin/streptomycin at 37°C in a humidified 5% CO_2_ incubator.

### Cell viability

Cell viability was measured by using the Cell Counting Kit-8 (CCK-8) assay according to the manufacturer’s instructions (Dojindo Molecular Technologies, Inc., Rockville, MD, USA). RAW 264.7 cells were seeded into a 96-well plate at a density of 5 × 10^4^ cells/well. After 24 h, the cells were treated with HCE (100 μg/mL) plus LPS (1 μg/mL), the HCE-Hx, HCE-MC, HCE-BuOH, or HCE-water fraction (100 μg/mL) plus LPS (1 μg/mL), the HCE-EA fraction (25, 50, 100, or 200 μg/mL) plus LPS (1 μg/mL), or LPS alone for an additional 24 h. The CCK-8 assay was employed after another 1 h to assess cell viability, and the absorbance at 450 nm was measured by using a Wallac EnVision™ Microplate Reader (PerkinElmer, MA, USA).

### Measurement of NO production

The nitrite concentration in the culture medium was measured as an indicator of NO production by the Griess reaction system (Promega, WI, USA). RAW 264.7 cells (5 × 10^4^ cells/well) in 96-well plates were cultured for 24 h. The cells were then treated with the samples list above plus LPS (1 μg/mL) or LPS alone for 20 h. The supernatant was mixed with the same volume of Griess reagent and incubated at room temperature for 5 min. The concentration of nitrite was determined by measuring the absorbance with a Wallac EnVision™ Microplate Reader (PerkinElmer).

### Determination of PGE_2_, TNF-α, and IL-6 production

RAW 264.7 cells (5 × 10^4^ cells/well in 96-well plates) were treated for 20 h with HCE plus LPS (1 μg/mL), the HCE-Hx, HCE-MC, HCE-BuOH, or HCE-water fraction (100 μg/mL) plus LPS (1 μg/mL), the HCE-EA fraction (25, 50, 100, 200 μg/mL) plus LPS (1 μg/mL), or LPS alone. The conditioned medium was then collected. The production of PGE_2_, TNF-α and IL-6 in the conditioned media was determined by using an ELISA kit (R&D systems) according to the manufacturer’s instructions.

### RNA extraction and quantitative real-time polymerase chain reaction (RT-PCR)

RAW 264.7 cells (5 × 10^5^ cells/well in six-well plates) were treated with HCE-EA (25, 50, 100, or 200 μg/mL) plus LPS (1 μg/mL) for 6 h. To evaluate the expression levels of iNOS and COX-2 mRNA, total cellular RNA was extracted with an RNeasy mini kit (Qiagen) according to the manufacturer’s instructions. Total RNA (500 ng) was mixed with Omniscript RT mixture (Qiagen) containing oligo-dT primers and water to a final volume of 20 μL and incubated at 37°C for 60 min. Real-time PCR was performed by using the Rotor Gene Q system (Qiagen, Hilden, Germany) and a reaction mixture that consisted of SYBR Green 2 × PCR Master Mix, cDNA template, and forward and reverse primers. The PCR protocol consisted of 35 cycles of denaturation at 95°C for 15 sec, followed by 55°C for 30 sec to allow for extension and amplification of the target sequence. The expression levels of iNOS and COX-2 mRNA were normalized to that of glyceraldehyde 3-phosphate dehydrogenase (GAPDH) via the 2- ΔΔCT method. The primer sequences employed in this study are shown in Table 1.

**Table 1:** Sequences of primers used in real-time PCR

**Gene**	**Forward**	**Reverse**	**Accession no.**	**Length (bp)**
iNOS	AAGGTCTACGTTCAGGACATC	AGAAATAGTCTTCCACCTGCT	NM_010927	187
COX-2	TTCCTCTACATAAGCCAGTGA	TCCACATTACATGCTCCTATC	NM_011198	200
GAPDH	TGTGTCCGTCGTGGATCTGA	CCTGCTTCACCACCTTCTTGA	NM_008084	77

### Preparation of cytosolic and nuclear extracts

RAW 264.7 cells (5 × 10^5^ cells/well in 6-well plates) were pretreated with HCE-EA (25, 50, 100, or 200 μg/mL) for 2 h, followed by stimulation with LPS (1 μg/mL) for 1 h. The preparation of nuclear and cytoplasmic extracts was performed by using the Nuclear Extract Kit (Active Motif). Lysates were collected and cleared by centrifugation, and the fractions were stored at -80°C prior to use.

### Western blotting analysis

RAW 264.7 cells (5 × 10^5^ cells/well in 6-well plates) were pretreated with HCE-EA (25, 50, 100, or 200 μg/mL) for 2 h, followed by stimulation with LPS (1 μg/mL). The cells were collected and washed twice with cold phosphate buffered saline (PBS). Total cellular proteins were extracted with a protein lysis buffer (Pro-prep, iNtRON, Sungnam, Korea). Equal amounts of protein (20 μg) were separated by sodium dodecyl sulfate-polyacrylamide gel electrophoresis (SDS-PAGE) in 12% gels, followed by transfer onto nitrocellulose membranes. Membranes were blocked with 5% non-fat milk and incubated with primary antibodies overnight at 4°C. The membranes were then incubated with the corresponding horseradish peroxidase-conjugated secondary antibodies for 1 h at room temperature. Membranes were treated with enhanced chemiluminescence detection reagents, and protein bands were visualized by using a Las-4000 Luminescent Image Analyzer (Fujifilm, Tokyo, Japan). Also, the densities of the bands were measured with the Multi Gauge software, version 3.0.

### Chromatographic conditions

Chromatographic analysis was performed with a ultra performance liquid chromatography (UPLC) system (Waters Co., Milford, MA, USA) and a photodiode array detector. HCE and HCE-EA samples (10 mg) were dissolved in methanol (10 mL). Chromatographic separation was then conducted by using an Acquity® UPLC HSS T3 column (2.1 × 100 mm, 1.8 μm, Waters Co). The mobile phase consisted of 0.2% acetic acid in water (A) and acetonitrile (B), and the gradient program encompassed a linear change in 0–12 min from 88:12 (v/v) to 75:25 (v/v). The detection wavelength was scanned at 210–400 nm and recorded at 254 nm. A flow rate of 0.2 mL/min was applied, and the sample injection volume was 1.0 μL.

### Instrumentation and analytical conditions

The liquid chromatography mass spectrometry (LC/MS) analysis was performed using an Waters UPLC system equipped with 15 T Fourier transform ion cyclotron resonance mass spectrometry (15 T FT-ICR MS) (Bruker, Billerica, MA) coupled with electrospray ionization (ESI). The mobile phase consisted of 0.1% formic acid in water (A) and 0.1% formic acid in acetonitrile (B) and the gradient program encompassed a linear change in 0–12 min from 88:12 (v/v) to 75:25 (v/v). Other LC/MS analysis condition was carried out in the same method with UPLC-PDA. Mass spectrometric acquisition was performed in scan mode using positive and mass range from *m/z* 150 to 2000. The ion source dependent parameters were: source accumulation, 0.020 sec; ion accumulation time, 0.400 sec; ion spray voltage floating 4500 V; dry Gas, 4.0 L/min; dry temperature, 180°C; nebulizer, 2.0 bar.

### Statistical analysis

All results are presented as the mean ± the standard deviation (SD) and are representative of three or more independent experiments. Data were compared by using Student’s *t*-test and *P*-values less than 0.05 were considered statistically significant.

## Results

### Effect of HCE and its associated fractions on cell viability and LPS-induced production of NO, PGE_2_, TNF-α, and IL-6

**Figure 1:**
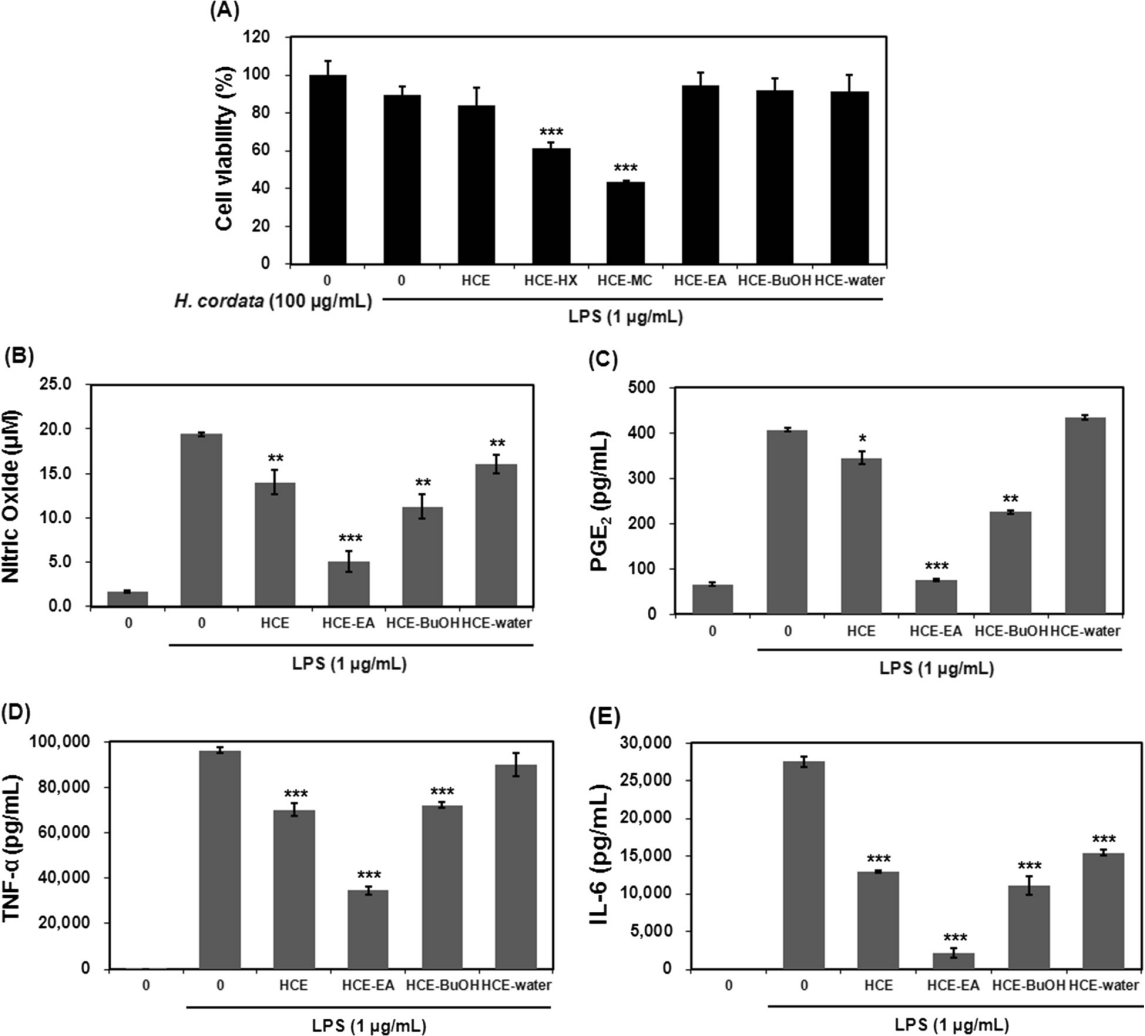
**Effect of *H. cordata *fractions on cell viability and the production of inflammatory mediators in LPS-stimulated RAW 264.7 macrophages. (A)** Cells were treated with HCE or *H. cordata* fractions (100 μg/mL) plus LPS (1 μg/mL) or LPS alone for 24 h. Cell viability was determined by using CCK-8 assay. **(B-E)** Cells were treated with various concentrations (25, 50, 100, or 200 μg/mL) of HCE-EA plus LPS (1 μg/mL) or LPS alone for 20 h. The production of **(B)** NO was measured by using the Griess reagent, and the production of **(C)** PGE_2_, **(D)** TNF-α, and **(E)** IL-6 was measured by using an ELISA kit. Values represent the mean ± the standard deviation (SD) (n = 3 independent experiments. *p < 0.05, **p < 0.01, ***p < 0.001 vs. LPS alone (control)).

The CCK-8 assay was employed to determine the effect of HCE and the HCE-Hx, HCE-MC, HCE-EA, HCE-BuOH, and HCE-water fractions on RAW 264.7 cell viability. As shown in Figure 1A, The HCE-Hx and HCE-MC fractions exhibited marked cytotoxicity toward the cells. Therefore, HCE-Hx and HCE-MC were excluded from the remaining experiments. Next, we investigated the impact of HCE, HCE-EA, HCE-BuOH, and HCE-water on LPS-stimulated production of NO, PGE_2_, TNF-α, and IL-6. Of these, HCE-EA was the most efficacious in regard to inhibition of the LPS-induced release of inflammatory mediators (Figure 1B-E). Thus, the HCE-EA fraction was selected for further study to assess the anti-inflammatory mechanism of *H. cordata*.

### Dose-dependent effect of HCE-EA on cell viability

**Figure 2:**
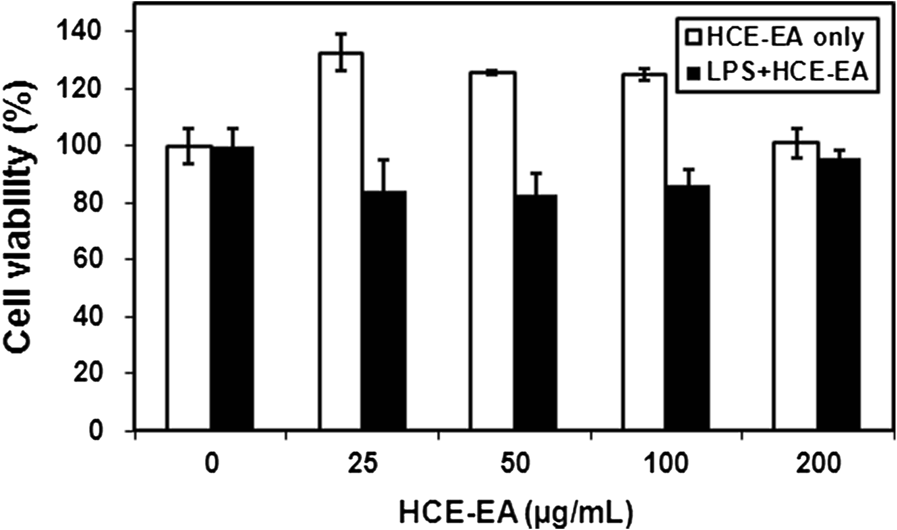
**Effect of HCE-EA on cell viability in RAW 264.7 cells.** Cell viability was measured by using the CCK-8 assay. Values represent the mean ± the SD (n = 3 independent experiments).

The influence of various concentrations of HCE-EA on RAW 264.7 cell viability was next investigated to establish the appropriate concentration range for the remainder of the study. HCE-EA did not affect cell viability at concentrations up to 200 μg/mL (Figure 2). Thus, we used HCE-EA at 25-200 μg/mL in subsequent experiments.

### Effect of HCE-EA on LPS-stimulated production of NO, PGE_2_, TNF-α, and IL-6

**Figure 3:**
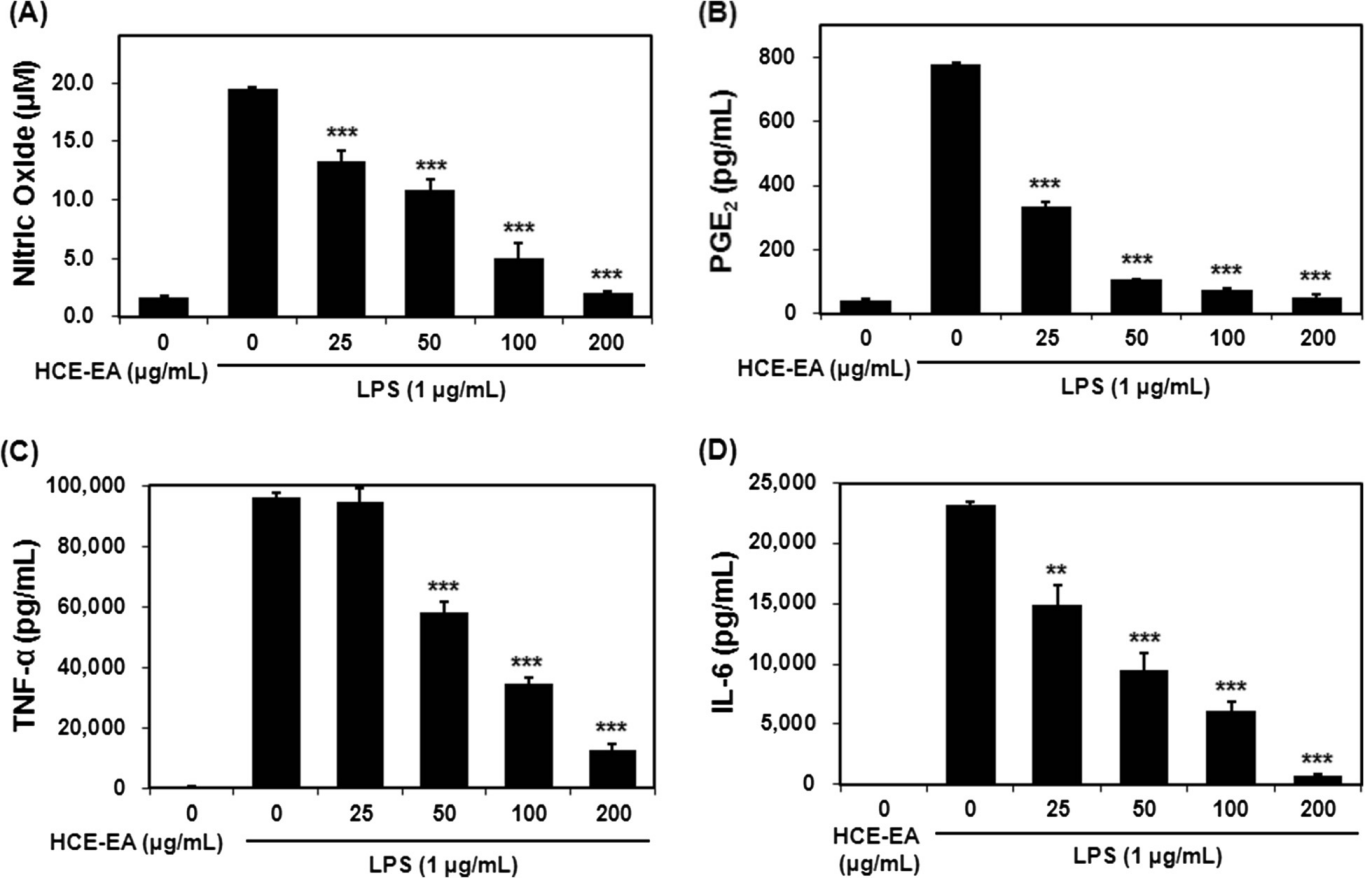
**Effect of HCE-EA on the production of inflammatory mediators in LPS-stimulated RAW 264.7 macrophages.** Cells were treated with various concentrations (0, 25, 50, 100 or 200 μg/mL) of HCE-EA plus LPS (1 μg/mL) or LPS alone for 20 h. **(A)** NO content in the conditioned medium was measured by using the Griess reagent. **(B-D)** PGE_2_, TNF-α, and IL-6 production were determined by using an ELISA kit. Each bar represents the mean ± the SD (n = 3 independent experiments **p < 0.01, ***p < 0.001 vs. LPS alone (control)).

To investigate whether HCE-EA inhibits LPS-stimulated production of NO, PGE_2_, TNF-α, and IL-6, cells were treated with various concentrations of HCE-EA plus LPS or LPS alone for 20 h. HCE-EA significantly decreased NO, PGE_2_, TNF-α, and IL-6 levels in a dose-dependent manner (Figure 3). The IC_50_ values of NO, PGE_2_, TNF-α, and IL-6 were estimated to be 58.64 ± 5.25, 22.12 ± 2.78, 70.99 ± 5.84, and 40.61 ± 1.54 μg/mL, respectively.

### Effects of HCE-EA on LPS-stimulated expression of iNOS and COX-2

**Figure 4:**
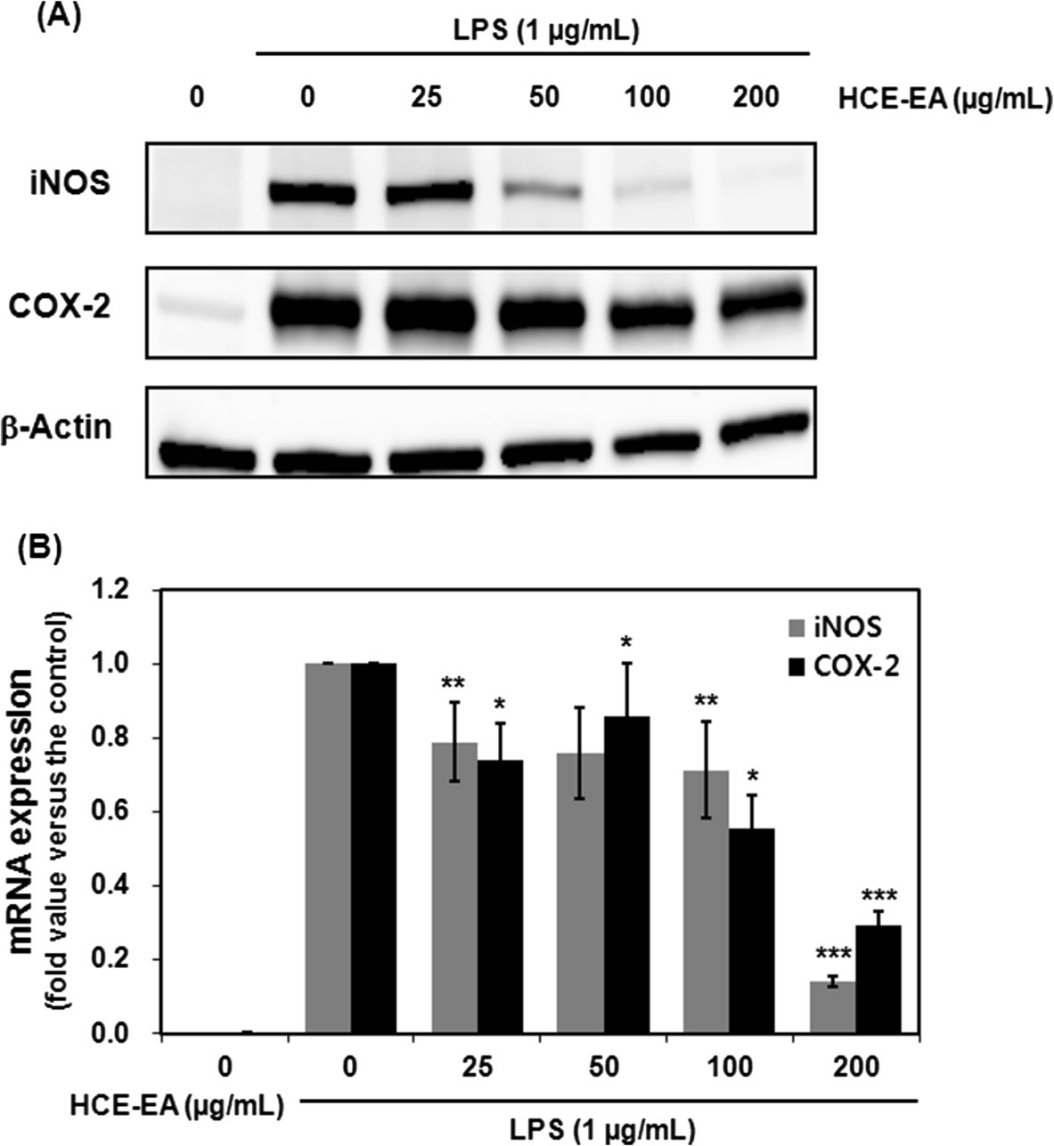
**Effect of HCE-EA on iNOS and COX-2 protein and mRNA expression in LPS-stimulated RAW 264.7 macrophages. (A)** Cells were treated with the indicated concentrations of HCE-EA plus LPS (1 μg/mL) or LPS alone for 20 h. Protein expression levels of iNOS and COX-2 were determined by Western blotting analysis. β-actin was employed as a loading control. **(B)** Cells were incubated with the indicated concentrations of HCE-EA LPS (1 μg/mL) or LPS alone for 6 h. mRNA expression levels of iNOS and COX-2 were determined by real-time PCR analysis. The expression level of GAPDH mRNA served as the internal control for the normalization of iNOS and COX-2 mRNA expression. Data are expressed as the mean ± the SD (n = 3 independent experiments *p < 0.05, **p < 0.01, ***p < 0.001 vs. LPS alone (control)).

Next, we investigated whether the inhibitory effect of HCE-EA on NO and PGE_2_ production was related to the modulation of iNOS and COX-2 protein levels. Figure 4A demonstrates that HCE-EA strongly suppressed the protein expression of both iNOS and COX-2. Furthermore, HCE-EA also significantly repressed iNOS and COX-2 mRNA expression in LPS-stimulated RAW 264.7 cells (Figure 4B). These results suggest that the HCE-EA-mediated inhibition of NO and PGE_2_ production is associated with transcriptional downregulation of iNOS and COX-2 genes.

### Effect of HCE-EA on LPS-induced NF-κB activation

**Figure 5:**
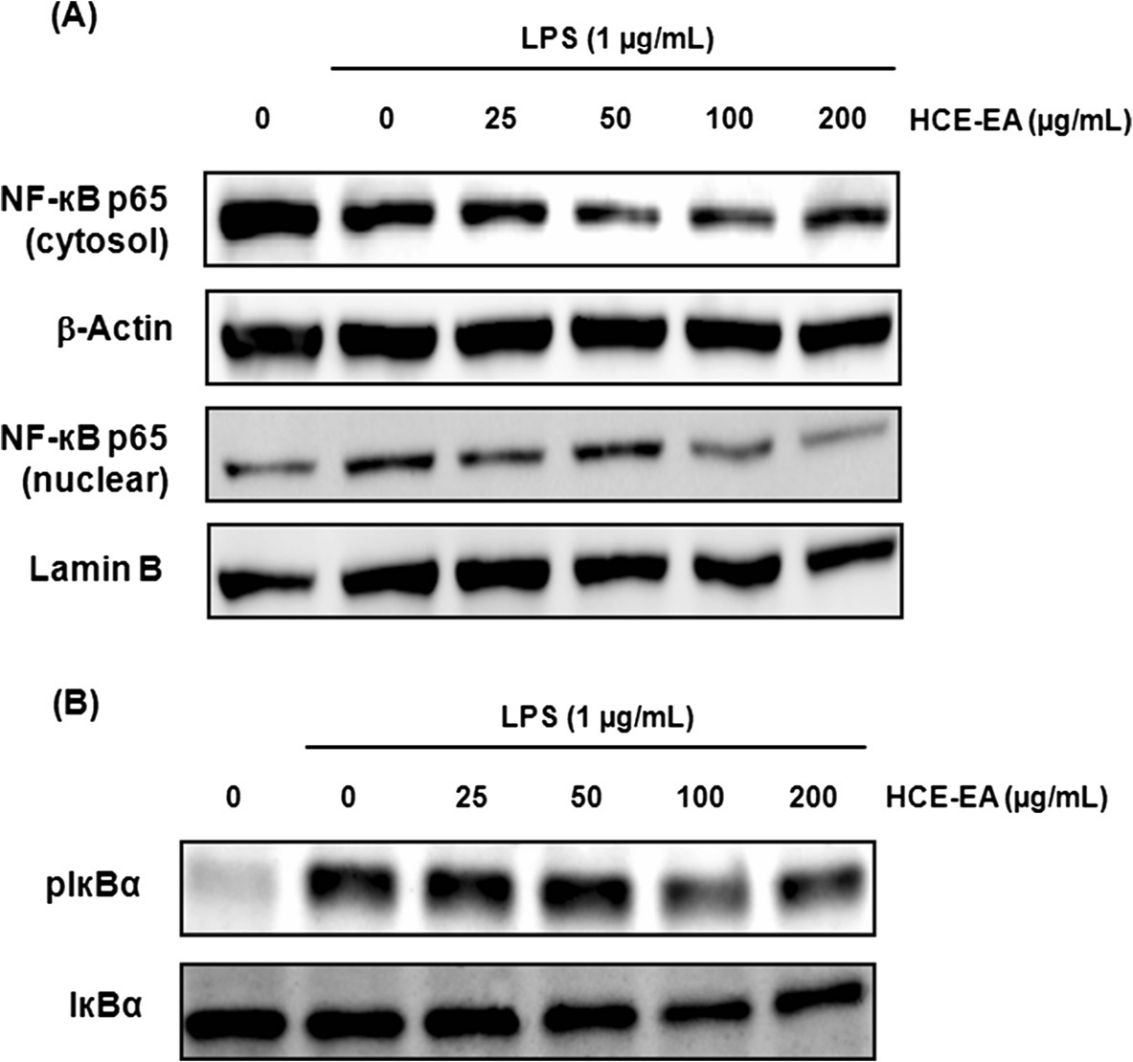
**Effect of HCE-EA on NF-κB activation in LPS-stimulated RAW 264.7 macrophages.** Cells were pretreated with HCE-EA (25, 50, 100, or 200 μg/mL) for 2 h prior to incubation with LPS (1 μg/mL) for another 1 h. Protein levels of **(A)** NF-κB p65 and **(B)** IκBα and pIκBα were determined by Western blotting analysis. Lamin B was employed as a loading control.

Activation of NF-κB, an essential transcription factor in the inflammatory response, occurs following the phosphorylation, ubiquitination, and proteolytic degradation of IκBα. Thus, p65 levels in cytoplasmic and nuclear extracts prepared from RAW 264.7 cells were next evaluated by Western blotting analysis. HCE-EA inhibited LPS-stimulated nuclear translocation of NF-κB p65 in a dose-dependent manner (Figure 5A). We also examined the ability of HCE-EA to inhibit the phosphorylation and degradation of IκBα in the cytoplasm and found that HCE-EA promoted the cytosolic accumulation of IκBα via suppression of IκBα phosphorylation (Figure 5B). These results suggest HCE-EA effectively inhibits LPS-induced NF-κB activation by blocking the nuclear translocation of NF-κB and the degradation of IκBα.

### Effect of HCE-EA on LPS-stimulated phosphorylation of MAPKs

**Figure 6:**
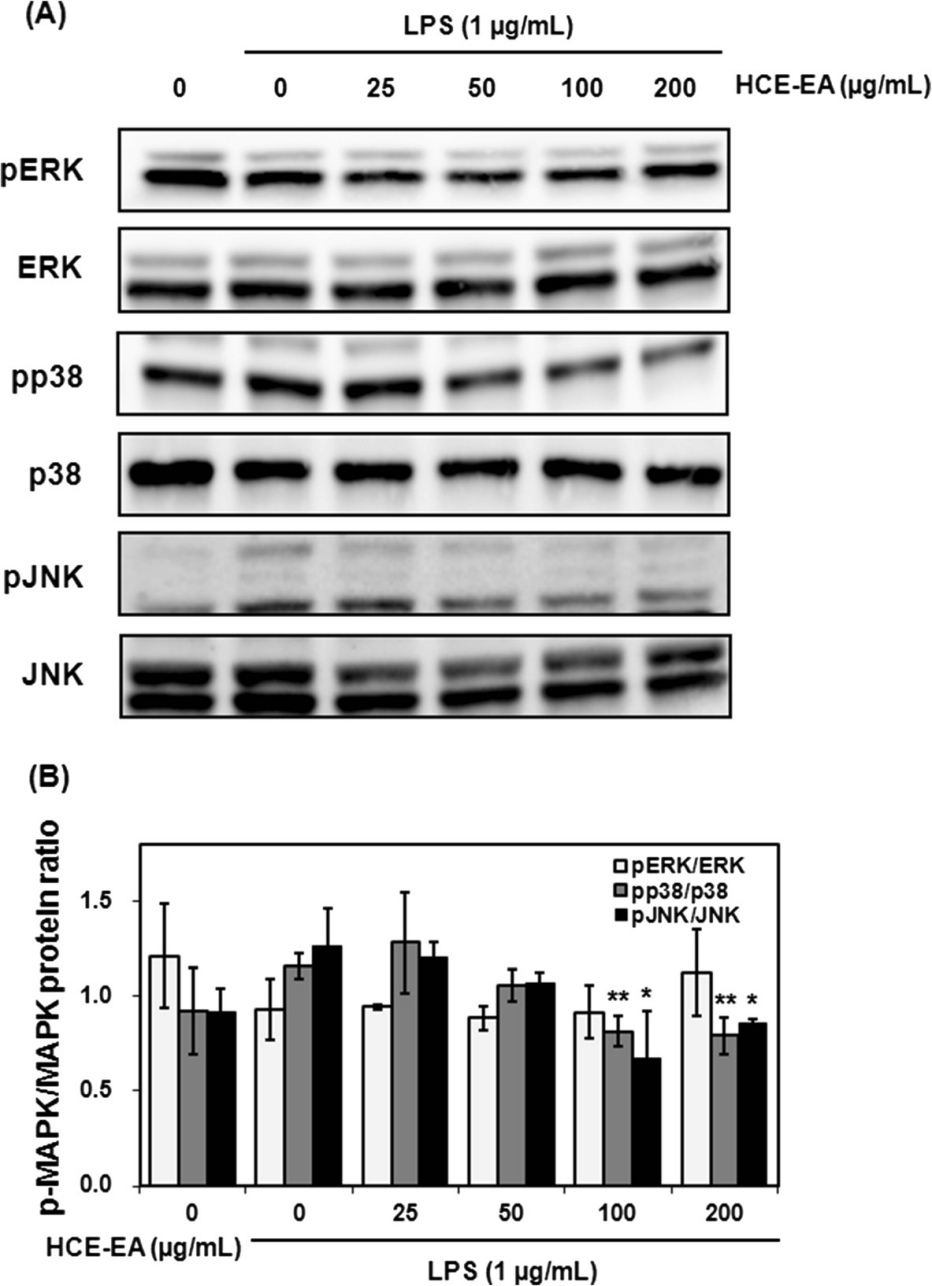
**Effect of HCE-EA on the phosphorylation of MAPKs in LPS-stimulated RAW 264.7 macrophages. (A)** Cells were pretreated with HCE-EA (25, 50, 100, or 200 μg/mL) for 2 h prior to incubation with LPS (1 μg/mL) for 30 min. Protein levels of ERK, pERK, JNK, pJNK, p38, and pp38 MAPKs were determined by Western blotting analysis. **(B)** The pMAPK/MAPK protein ratio in shown. Values represent the mean ± the SD (n = 3 independent experiments *p < 0.05, **p < 0.01, vs. LPS alone (control)).

The phosphorylation and activation of MAPKs are crucial for LPS-stimulated NF-κB activation and the subsequent activation of inflammatory mediators [25]. To investigate whether HCE-EA attenuates inflammatory responses through MAPK pathways, we employed Western blotting to evaluate the phosphorylation levels of several MAPKs, including extracellular signal regulated kinase (ERK) 1/2, c-Jun N-terminal kinase (JNK), and p38. HCE-EA suppressed the LPS-induced phosphorylation of JNK and p38, but not ERK 1/2 (Figure 6). This observation is consistent with the hypothesis that HCE-EA blocks JNK and p38 phosphorylation in MAPK pathways to suppress inflammatory responses in LPS-induced RAW 264.7 cells.

### Identification of the primary active components in HCE-EA

**Figure 7:**
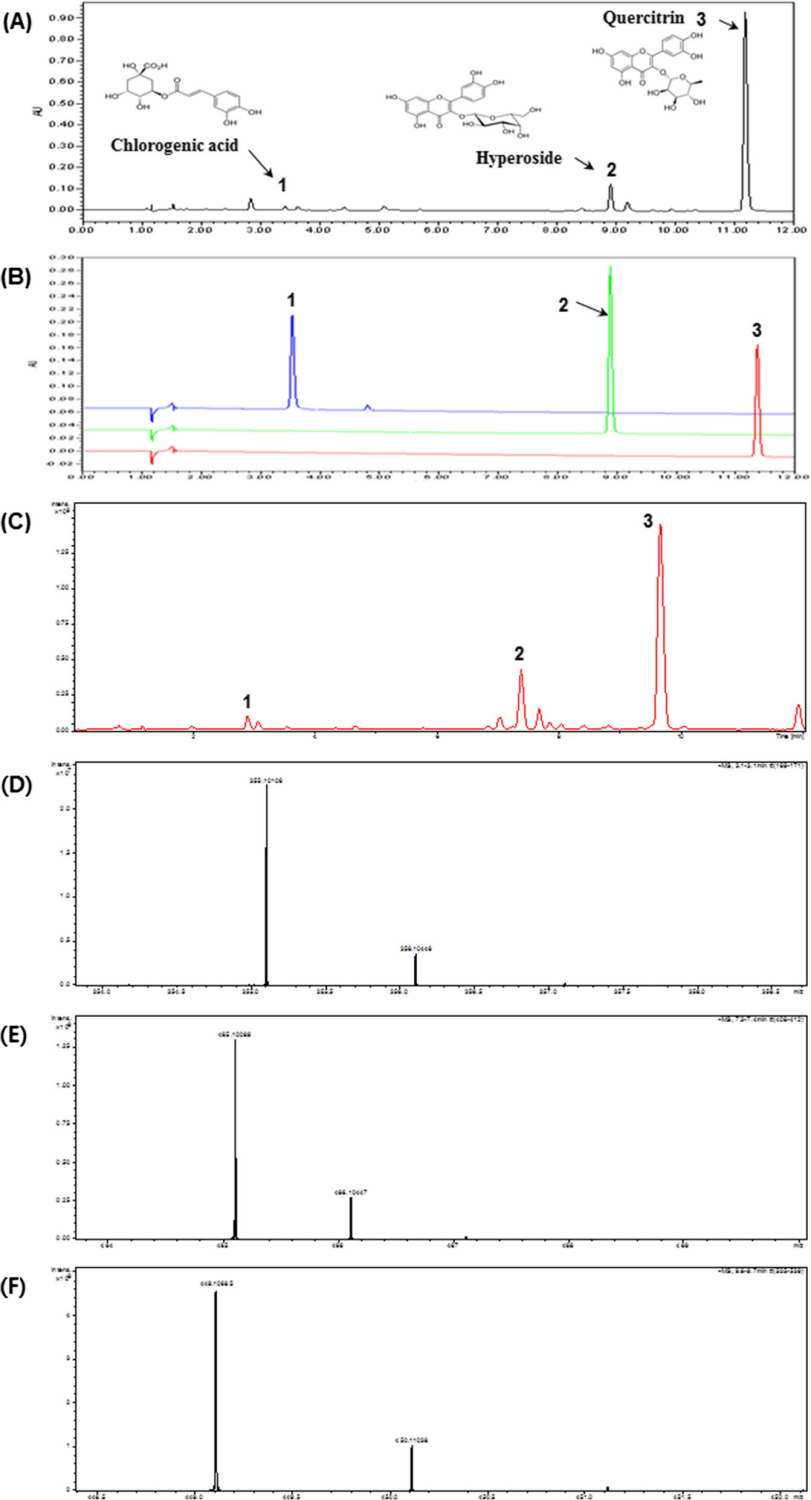
**Representative UPLC chromatogram and mass spectra. (A)** HCE-EA, **(B)** Peaks represent of standard compounds: (1) chlorogenic acid, (2) hyperoside, and (3) quercitrin, **(C)** total ion chromatogram of HCE-EA, **(D)** mass spectra of chlorogenic acid, **(E)** mass spectra of hyperoside, **(F)** mass spectra of quercitrin.

UPLC-PDA and LC/MS analysis was next performed to identify the primary functional compounds in HCE-EA. The chromatogram and mass spectra of three components (chlorogenic acid, hyperoside, and quercitrin) are shown in Figure 7. The three components in HCE-EA were detected at approximately 3.4, 8.9 and 11.3 min, respectively (Figure 7A and B). The molecular weight of three components was confirmed by LC/MS and their mass spectra. The product ion scan spectra of [M + H]^+^ for chlorogenic acid, hyperoside, and quercitrin, showed molecular peak at *m/z* 355.10, 465.10, and 449.10, respectively (Figure 7C-F). As shown in Table 2, three components were increased in HCE-EA compared with HCE and are thus candidates for the anti-inflammatory actions of HCE-EA.

**Table 2:** Content of three compounds in HCE and HCE-EA

**Material**	**Content (mg/g)**		
	**Chlorogenic acid**	**Hyperoside**	**Quercitrin**
HCE	1.052 ± 0.00	4.342 ± 0.02	8.999 ± 0.03
HCE-EA	12.503 ± 0.02	38.705 ± 0.07	142.671 ± 0.24

## Discussion

Inflammatory processes are distinguished by the generation of large amounts of the pro-inflammatory mediators NO and PGE_2_, which are in turn generated by iNOS and COX-2 enzymes, respectively [26]. NO signaling requires iNOS upregulation, whereas abundant COX-2 expression promotes PGE_2_ production and the activation of the pro-inflammatory PGE_2_ signaling cascade in response to inflammatory stimuli [27]. Pro-inflammatory cytokines (e.g., TNF-α and IL-6) evoke elevated levels of iNOS and COX-2, followed by a significant increase in NO and PGE_2_ production. Therefore, the present study evaluated the anti-inflammatory actions of HCE-EA on LPS-stimulated RAW 264.7 cells and the molecular mechanisms involved in terms of TNF-α, IL-6, iNOS, COX-2, NO, and PGE_2_ production.

The current results showed that HCE-EA was more efficacious than the other fractions at suppressing the LPS-induced release of NO and PGE_2_, with no cytotoxicity at the concentrations employed. Moreover, HCE-EA suppressed the LPS-stimulated increase in iNOS and COX-2 protein and mRNA expression. We also confirmed that HCE-EA decreased the levels of pro-inflammatory cytokines produced by LPS-treated RAW 264.7 cells (i.e., TNF-α and IL-6), and showed that the actions of HCE-EA were mediated via inhibition of the NF-κB and MAPK signaling pathways. These pathways are well-known to modulate levels of pro-inflammatory mediators.

NF-κB regulates the transcription of a number of genes, including iNOS, COX-2, TNF-α, and IL-6, and is thus important for the development of inflammatory diseases [7,12,14]. Once activated, the NF-κB p65 subunit dissociates from its inhibitory protein IкBα and translocates from the cytoplasm into the nucleus [28]. We demonstrated herein that HCE-EA inhibits NF-κB activation via the blockade of LPS-induced IкBα degradation and the subsequent nuclear translocation of the NF-κB p65 subunit. MAPK signaling pathways are also directly involved in the synthesis of pro-inflammatory cytokines in activated macrophages through the induction of NF-κB [29,30]. Therefore, NF-κB- and MAPKs- targeted therapeutics might be effective for the treatment of inflammatory diseases, given that a wide variety of pharmacologic agents reportedly inhibit activation steps in the NF-κB and MAPKs signaling pathways [13,31,32]. Consistent with these reports, the current study demonstrated that HCE-EA dose-dependently inhibited the phosphorylation of p38 and JNK MAPKs. These results suggest that the HCE-EA-mediated inhibition of NF-κB and MAPK activations is related to the reduced production of pro-inflammatory mediators in LPS-stimulated RAW 264.7 cells.

This study showed that the levels of three compounds (chlorogenic acid, hyperoside, and quercitrin) were elevated in HCE-EA compared with HCE. A previous study reported that chlorogenic acid exerted as anti-inflammatory actions by inhibiting the LPS-provoked release of inflammatory cytokines in RAW 264.7 cells [33]. Additional studies demonstrated that hyperoside inhibited NO production in rat peritoneal macrophages and attenuated LPS-induced inflammatory responses through NF-κB activation and IκB-α degradation [34,35]. Yet another study revealed that quercitrin markedly overturned NO production in RAW 264.7 cells and inhibited the progression of inflammation through down-regulation of the NF-κB pathway [36,37]. Furthermore, Tian et al [15] reported that an EA fraction derived from *H. cordata* was rich in the polyphenolic compounds quercitrin, quercetin, and hyperoside, which were responsible for the antioxidant and hepatoprotective merits of the tea. In agreement with these previous studies, our results indicate that the ability of HCE-EA to suppress production of inflammatory mediators might be attributable to its content of active components, in particular chlorogenic acid, hyperoside, and quercitrin.

## Conclusions

In conclusion, HCE-EA inhibited the production of NO, PGE_2_, iNOS, COX-2, TNF-α, and IL-6 in LPS-treated RAW 264.7 cells. The inhibitory effects were mediated by inhibition of NF-κB activation and MAPK (p38 and JNK) signaling pathways. Thus, HCE-EA may find utility as an attractive agent to prevent or reverse inflammatory responses.

## Competing interests

The authors declare that they have no competing interest.

## Authors’ contributions

JMC, KJN and HKK participated in the design of the study data analyses and manuscript preparation. HSK, AYL, BCM carried out the *Houttuynia cordata* fractionation and analyzed the HPLC data. All authors read and approved the final manuscript.

## Pre-publication history

The pre-publication history for this paper can be accessed here:

http://www.biomedcentral.com/1472-6882/14/234/prepub
